# Risk factors for collisions attributed to microsleep-related behaviors while driving in professional truck drivers

**DOI:** 10.1038/s41598-024-57021-1

**Published:** 2024-03-16

**Authors:** Hiroyuki Sawatari, Hajime Kumagai, Kengo Kawaguchi, Yuka Kiyohara, Noriyuki Konishi, Aki Arita, Mitsuo Hayashi, Toshiaki Shiomi

**Affiliations:** 1 Department of Perioperative and Critical Care Management, Graduate School of Biomedical and Health Sciences, Hiroshima University, Hiroshima, Japan; 2 Department of Sleep Medicine, Graduate School of Biomedical and Health Sciences, Hiroshima University, 1-2-3 Kasumi, Minami-Ku, Hiroshima, 7348533 Japan; 3 Sleep Disorders Center, Hiroshima University Hospital, Hiroshima, Japan; 4 Graduate School of Integrated Arts and Sciences, Hiroshima University, Hiroshima, Japan

**Keywords:** Health care, Health occupations, Medical research, Risk factors, Signs and symptoms

## Abstract

Sleep-disordered breathing (SDB) is prevalent among professional drivers. Although SDB is a known risk factor for truck collisions attributed to microsleep-related behaviors at the wheel (TC-MRBs), the usefulness of overnight pulse oximetry for predicting TC-MRBs is debatable. This retrospective study assessed the association between overnight pulse oximetry parameters, the Epworth Sleepiness Scale (ESS), and TC-MRBs, confirmed by dashcam footage. This study included 108 matched professional truck drivers (TC-MRBs: N = 54; non-TC-MRBs: N = 54), with a mean age and body mass index of 41.9 ± 11.3 years and 23.0 ± 3.7 kg/m^2^, respectively. Night-time drivers, 4% oxygen desaturation index (ODI), and nadir oxygen saturation (SpO_2_) were associated with TC-MRBs (odds ratio [95% confidence interval]: 25.63 [5.88–111.77], p < 0.0001; 2.74 [1.02–7.33], p = 0.045; and 3.87 [1.04–14.39], p = 0.04, respectively). The area under the curve of 4% ODI and nadir SpO_2_ for TC-MRBs were 0.50 and 0.57, respectively. In conclusion, night-time driving, 4% ODI, and nadir SpO_2_ were significantly associated with TC-MRBs in professional truck drivers. However, the sensitivity of overnight pulse oximetry parameters to predict TC-MRBs in a real-world application was poor. Therefore, combining subjective and objective assessments such as dashcam video footage may be needed to achieve high accuracy for predicting TC-MRBs among professional truck drivers.

## Introduction

Sleep-disordered breathing (SDB), characterized by repetitive intermittent hypoxemia, overactivated sympathetic nerve activity, and arousal from sleep due to airway obstruction or the absence of respiratory effort, is common in the general population, with an estimated prevalence of 15%–50%^1–4^. Thus, SDB is a well-known risk factor for excessive daytime sleepiness (EDS), cognitive dysfunction, cardiovascular diseases, dementia, and metabolic disorders^2,5–7^.

Among professional drivers, the prevalence of SDB is known to range from 40.1 to 71.8%^8–10^. Previous studies on professional drivers have reported that the presence of SDB is associated with hypertension, cardiovascular diseases, and metabolic syndrome^9,11^. It has also been reported that poor sleep associated with SDB leads to psychological disorders, such as anxiety and depression, in professional drivers^12^. These studies indicate management of SDB in professional drivers would be important for their health. In addition to the adverse effects of SDB on physiological and psychological aspects, previous studies have reported a significant association between SDB, including EDS, and motor vehicle accidents (MVAs) in the general population^13,14^. Continuous positive airway pressure (CPAP) is the first-line treatment for SDB and can significantly reduce cardiovascular events and MVAs in patients with SDB^15–17^. However, conducting full polysomnography (PSG) screenings on all employees to assess SDB among professional drivers may be difficult, especially for large companies. Therefore, overnight pulse oximetry and the Epworth Sleepiness Scale (ESS) are frequently used. Nevertheless, the usefulness of using only overnight pulse oximetry for predicting an increased risk of MVAs attributed to falling asleep at the wheel is unknown, with oxygen saturation (SpO_2_) alone having a moderate accuracy for classifying SDB severity. Furthermore, the accuracy of overnight oximetry for monitoring SDB is also debatable^18,19^. In contrast, two previous studies reported a significant association between overnight pulse oximetry parameters and MVAs^20,21^. These studies indicate that the usefulness of assessing the risk of MVAs attributed to falling asleep at the wheel using overnight pulse oximetry is still unclear. Moreover, regarding self-report questionnaires which are frequently used in health checks, a previous study showed that professional truck drivers intentionally underreported sleepiness on questionnaires in order to maintain their driving licenses^22^.

The current methods for assessing the occurrence of falling asleep at the wheel are limited as they rely on self-reported sleepiness to distinguish truck collisions attributed to falling asleep at the wheel from other types of truck collisions, such as those caused by looking aside while driving. However, our previous study, which analyzed truck collisions based on behavioral assessments using dashcam video footage, showed that truck collisions by professional truck drivers were due to microsleep episodes at the wheel, indicating that early diagnosis of microsleep with behavioral assessments is crucial in preventing truck collisions attributed to falling asleep at the wheel^23^.

Thus, in this study, using a large nationwide database with dashcam video footage, we retrospectively assessed whether overnight pulse oximetry parameters and subjective reports of sleepiness were associated with truck collisions attributed to microsleep-related behaviors (TC-MRBs) among professional truck drivers.

## Results

**Figure 1:**
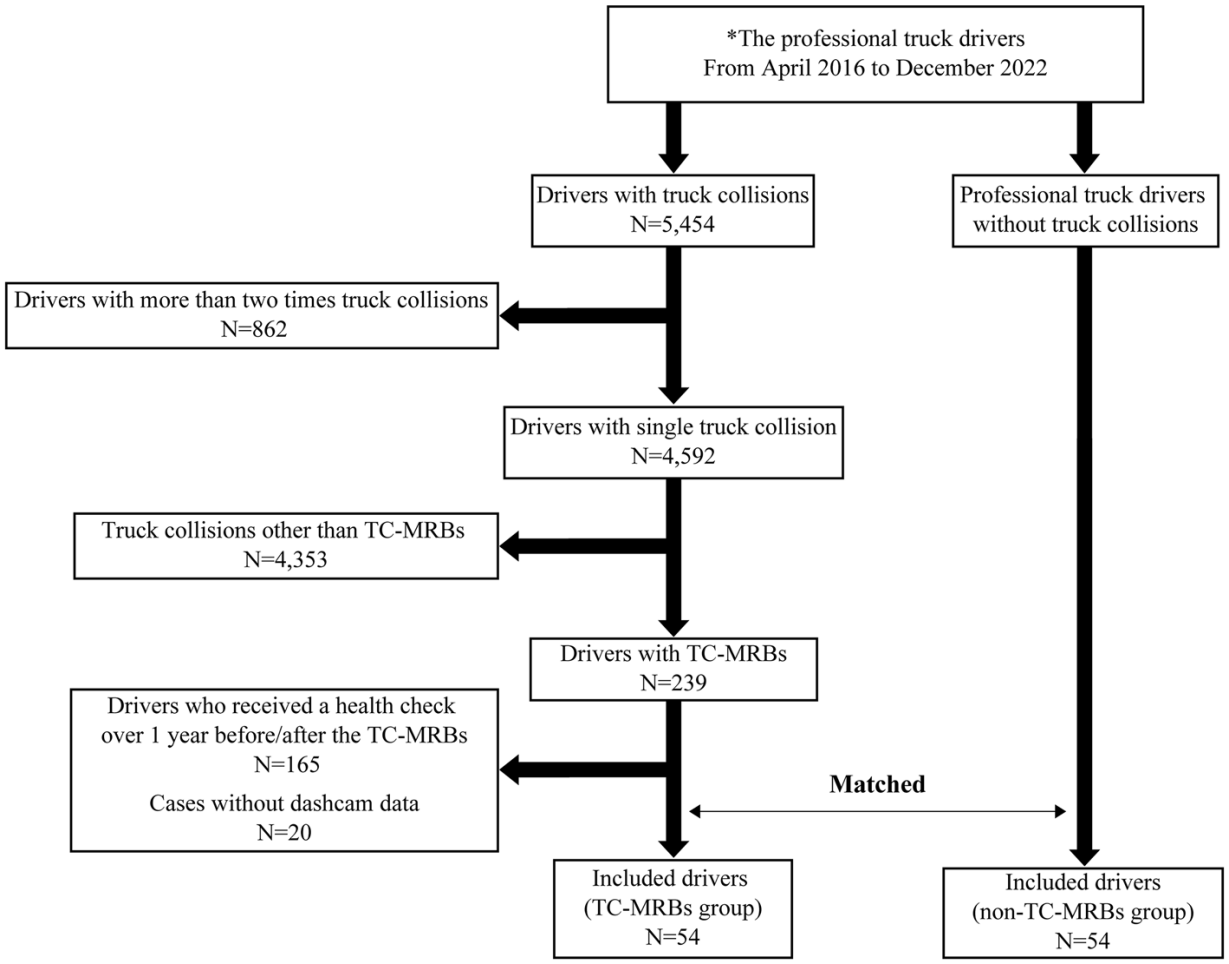
Study flow chart. *N* number, *TC-MRBs* truck collisions attributed to microsleep-related behaviors at the wheel.

The average number of professional truck drivers per year was 14,281 (see Supplementary Table 1). A total of 5,454 drivers experienced MVAs during the study period. Of the 4,592 professional truck drivers with single truck collisions (see Supplementary Table 2), 4,538 were excluded from the analysis based on the exclusion criteria (Fig. 1), leaving 54 professional truck drivers involved in TC-MRBs as confirmed by dashcam video footage (the TC-MRBs group).

Of the included professional truck drivers, the mean (± standard deviation, SD) age and body mass index (BMI) were 41.9 ± 11.3 years and 23.0 ± 3.7 kg/m^2^, respectively (Table 1). All truck drivers were male, and 33 of them (30.6%) worked the night shift. The average time between the health check and TC-MRB in the TC-MRBs group was 56.1 days. When comparing the TC-MRBs and non-TC-MRBs groups, no significant differences were observed in age, sex, BMI, or systolic and diastolic blood pressure. In contrast, the rate of night-time drivers in the TC-MRBs group was significantly higher than that in the non-TC-MRBs group (53.7 vs. 7.4%, p < 0.001).

**Table 1:** Characteristics of professional truck drivers.

	All	TC-MRBs	non-TC-MRBs	P-value	Cohen's d
Number, N	108	54	54	–	–
Age, years	41.9 ± 11.3	42.0 ± 11.2	41.7 ± 11.5	0.90	− 0.02
Male, N (%)	108 (100.0)	54 (100.0)	54 (100.0)	1.00	–
BMI, kg/m^2^	23.0 ± 3.7	23.8 ± 3.6	22.3 ± 3.7	0.052	− 0.40
Night-time drivers, N (%)	33 (30.6)	39 (53.7)	4 (7.4)	< 0.0001	–
SBP, mmHg	125.9 ± 13.2	125.6 ± 13.4	126.3 ± 13.1	0.80	0.05
DBP, mmHg	76.6 ± 10.5	76.3 ± 10.5	77.0 ± 10.5	0.72	0.07
Day differences between health check and TC-MRBs, days	–	56.1 ± 42.4	–	–	–

TC- MRBs: Truck collisions attributed to microsleep-related behaviors at the wheel; N: Number, BMI: Body mass index; SBP: Systolic blood pressure; DBP: Diastolic blood pressure.

**Figure 2:**
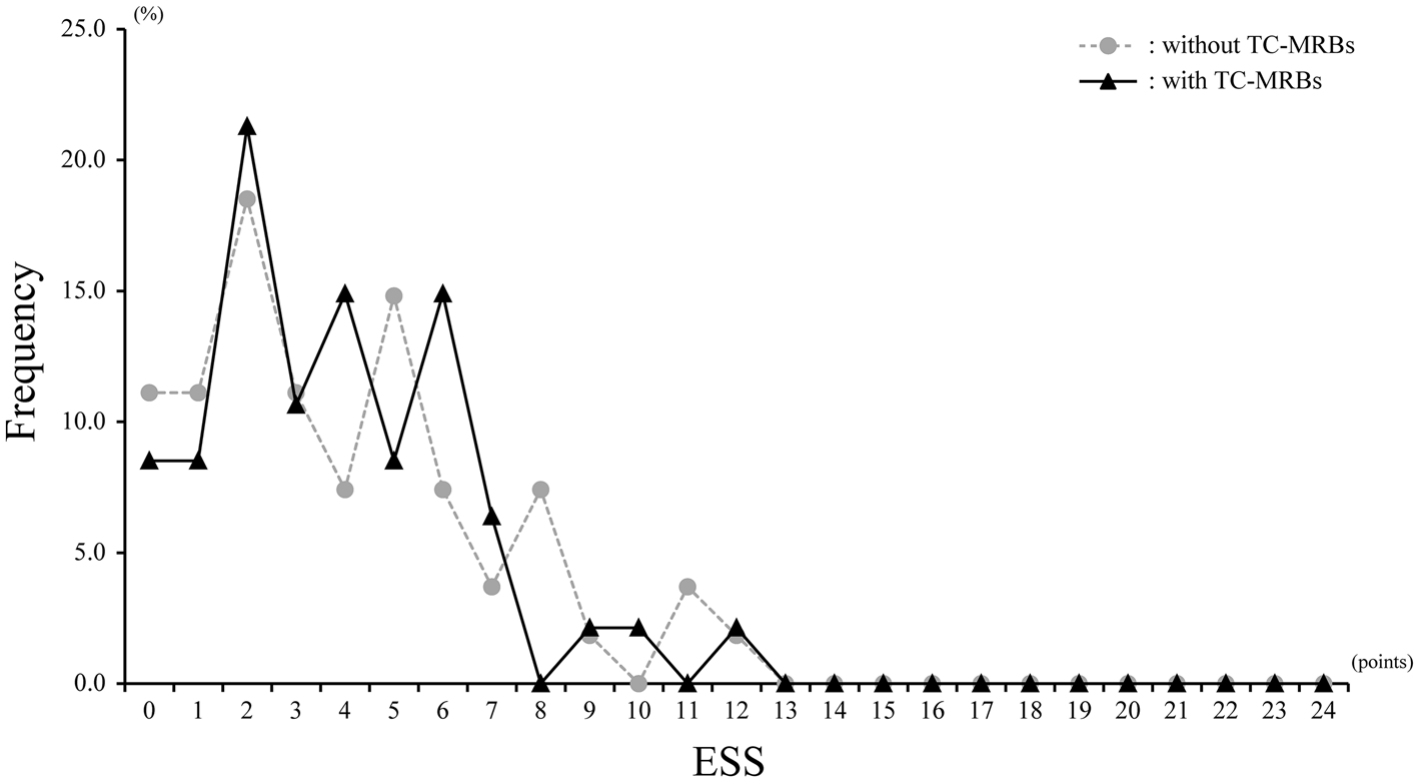
Trends of the Epworth Sleepiness Scale. ESS: Epworth Sleepiness Scale; TC-MRBs: Truck collisions attributed to microsleep-related behaviors at the wheel.

**Figure 3:**
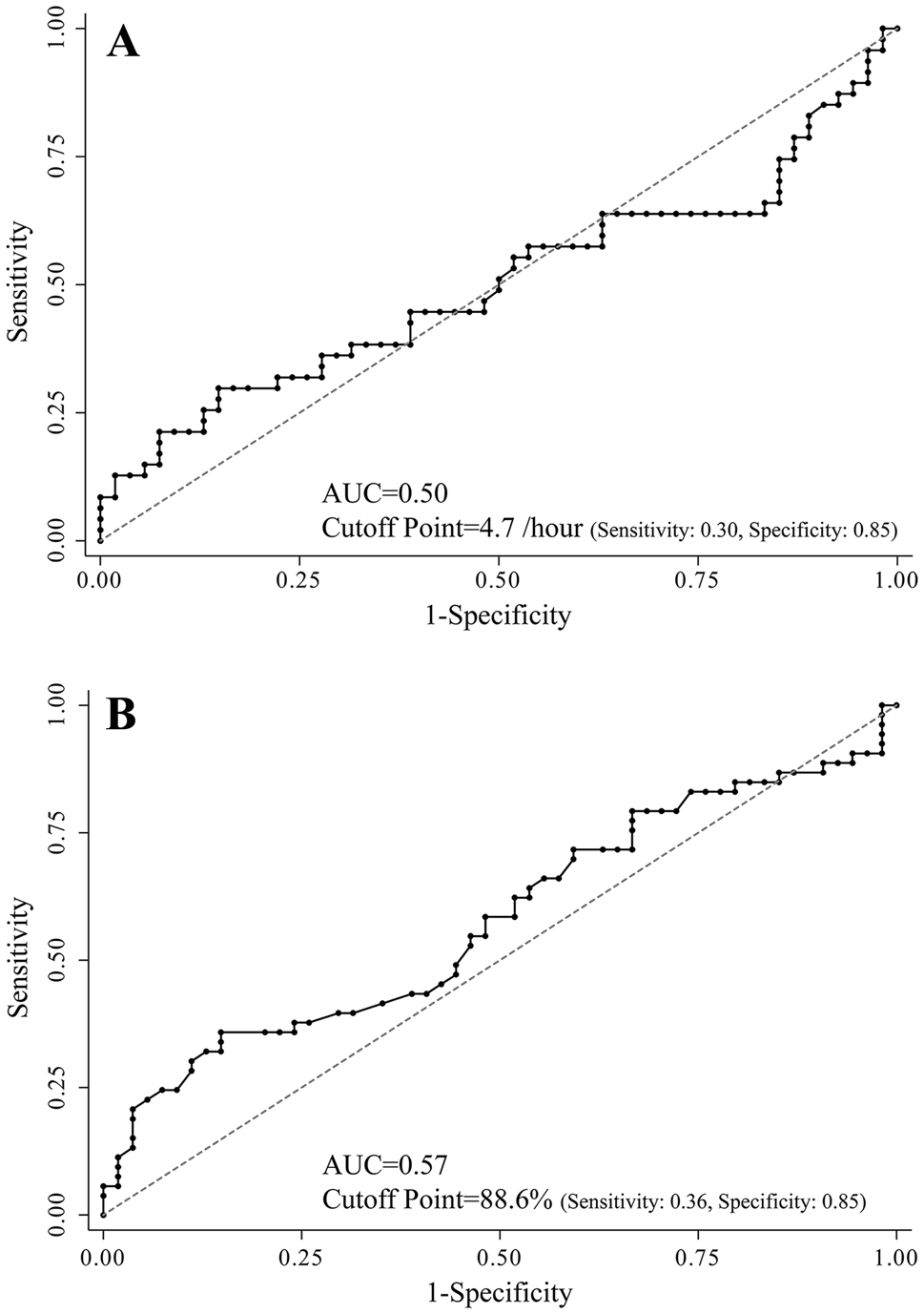
Receiver operating characteristic curves for truck collisions. (**A**) 4% oxygen desaturation index (ODI). (**B**) Nadir oxygen saturation (SpO_2_). AUC: Area under the curve.

There were no significant differences in daytime sleepiness, as indicated by the ESS scores, presence of EDS (Table 2, Fig. 2), 3% oxygen desaturation index (ODI), 4% ODI, nadir SpO_2_ values, and probable severity of SDB, between the two groups. Although the mean SpO_2_ in the TC-MRBs group was significantly lower than that in the non-TC-MRBs group (96.1 ± 1.1 vs. 96.4 ± 1.9%, p = 0.047), the actual effect size was small (Cohen's d: 0.28) (Table 2). In the stepwise logistic regression analysis, BMI, 3% ODI, mean SpO_2_, and ESS were not selected as model variables. However, night-time drivers, 4% ODI, and nadir SpO_2_ were significantly associated with TC-MRBs in professional truck drivers (odds ratio [OR] [95% confidence interval (95% Cl)] 25.63 [5.88–111.77], p < 0.0001; 2.74 [1.02–7.33], p = 0.045; and 3.87 [1.04–14.39], p = 0.04, respectively) (Table 3). In addition to the multivariate analysis, the area under the curve (AUC) of 4% ODI and nadir SpO_2_ for TC-MRBs were 0.50 [95% CI 0.38–0.62] and 0.57 [95% CI 0.47–0.69], respectively. The cutoff values of 4% ODI and nadir SpO_2_ for predicting TC-MRBs were estimated at 4.7/h (Fig. 3A) and 88.6%, respectively (Fig. 3B) (4% ODI: sensitivity = 0.30, specificity = 0.85; nadir SpO_2_: sensitivity = 0.36, specificity = 0.85).

**Table 2:** Differences of sleep related parameters.

	All	TC-MRBs	non-TC-MRBs	P-value	Cohen's d
SDB related parameters					
3%ODI, /h	6.4 ± 6.7	7.8 ± 8.8	5.0 ± 3.2	0.63	− 0.42
4%ODI, /h	3.9 ± 4.5	4.6 ± 6.1	3.2 ± 3.1	0.98	− 0.32
Nadir SpO_2_, %	83.9 ± 9.8	85.2 ± 6.1	82.7 ± 12.3	0.16	− 0.25
Mean SpO_2_, %	96.3 ± 1.1	96.1 ± 1.1	96.4 ± 1.9	0.047	0.28
*Probable severity of SDB					
Non	60 (55.6)	28 (51.9)	32 (59.3)		
Mild	40 (37.0)	19 (35.2)	21 (38.9)		
Moderate	4 (3.7)	3 (5.6)	1 (1.9)		
Severe	4 (3.7)	4 (7.4)	0 (0.0)	0.15	–
Daytime sleepiness					
ESS, points	3.9 ± 2.9	3.9 ± 3.0	3.9 ± 2.7	0.93	0.03
EDS, N (%)	4 (4.0)	1 (2.1)	3 (5.6)	0.38	–

*TC-MRBs* truck collisions attributed to microsleep-related behaviors at the wheel, *SDB* Sleep-disordered breathing, *ODI* Oxygen desaturation index, *ESS* The Epworth Sleepiness Scale, *EDS* excessive daytime sleepiness.

*The categorises were based on 3%ODI (non: 5 < times/h, mild: 5.0–14.9 times/h, moderate: 15.0–29.9 times/h, severe: ≥ 30.0 times/h).

We considered a driver to have EDS when the ESS score of the driver was over 10 points.

**Table 3:** Associated factors for truck collisions attributed to microsleep-related behaviors at the wheel in professional truck drivers.

	OR (95% CI)	P-value
Age	0.94 (0.89–1.00	0.04
BMI	Removed	0.81
Night-time drivers	25.63 (5.88–111.77)	< 0.0001
3%ODI	Removed	0.24
^¶^4%ODI	2.74 (1.02–7.33)	0.045
^¶^Nadir SpO_2_	3.87 (1.04–14.39)	0.04
Mean SpO_2_	Removed	0.20
ESS	Removed	0.38
*EDS	N/A	N/A

The variables included were age, BMI, working time zone, 3% ODI, 4% ODI, nadir SpO_2_, mean SpO_2_, ESS, and presence of EDS.

We considered a driver to have EDS when the ESS score of the driver was over 10 points.

*OR* odds ratio, *CI* confidence interval, *BMI* body mass index, *ODI* oxygen desaturation index, *ESS* The Epworth Sleepiness Scale, *EDS* excessive daytime sleepiness.

^¶^ OR and 95%CI were calculated as a change of one standard deviation.

*We cannot calculate because of few numbers of driver with EDS.

## Discussion

This large, real-world nationwide database study showed that TC-MRBs, verified by dashcam video footage, were not significantly associated with BMI, 3% ODI, mean SpO_2_, ESS scores, or the presence of EDS. However, the 4% ODI and nadir SpO_2_ showed significant associations with TC-MRBs in professional truck drivers. The receiver operating characteristic (ROC) curve analysis showed low AUC values with 4% ODI and nadir SpO_2_ being poor at identifying TC-MRBs, whereas night-time driver was strongly associated with TC-MRBs in the professional driver.

It is well-known that high BMI in professional drivers is a significant risk factor for the presence of SDB, whereas in this study, although the BMI of the study population was lower than that in previous studies, the prevalence of suspected SDB was 44.4%, which corresponds with previous studies on professional drivers^8–10^. One of the possible reasons is the differences in craniofacial structures among the ethnic groups. Previous studies reported that the cranial base, which was associated with the presence and severity of SDB, was significantly shorter in Asians than in Caucasians^24^. These studies indicate that the prevalence of SDB might not be significantly different among ethnicities despite the BMI being low in Asians. The facts indicate a regular screening for SDB might be needed to prevent TC-MRB even if the BMI in the professional driver is not high.

Many studies have assessed the relationship between SDB and sleepiness in professional truck drivers. Some studies reported that SDB could be a risk factor for MVAs among professional drivers^25,26^, while another study showed the opposite^27^. Although the definition of SDB in these studies was based on a self-report questionnaire, the results highlighted the potential difficulties of using this assessment for predicting MVAs. For overnight pulse oximetry parameters, our study found a significant association between 4% ODI/nadir SpO_2_ and TC-MRBs. This finding aligns with that of a previous study demonstrating a similar association between overnight pulse oximetry parameters and MVAs in bus drivers, while the low AUC values in our study indicates that 4% ODI and nadir SpO_2_ were poor at identifying TC-MRBs^20^.

Although previous studies have shown an association between MVAs and severe hypoxemia and EDS^28–30^, our study only showed a significant association between TC-MRBs and nadir SpO_2_, but not with ESS or EDS. As mentioned above, professional truck drivers tend to underreport their ESS/EDS to maintain their driving licenses^22^, which can hamper the prediction of collisions attributed to falling asleep at the wheel or TC-MRBs using ESS/EDS. Additionally, professional truck drivers usually drive longer distances and for longer times than general drivers. Notably, most professional truck drivers who have been involved in a TC-MRB drive without experiencing any drowsiness after extended distances/periods of time. This could be attributed to the fact that only a few drivers in this study had an ESS score of 11 or higher. However, during long-distance or extended-time driving, TC-MRBs occur when the truck drivers experience microsleep episodes lasting a few minutes or even seconds. Moreover, TC-MRBs have also been shown to surge just a few seconds before collisions^23^. Previous studies also reported that ESS alone may not be a suitable tool for detecting MVAs^31^. Similarly, our findings suggest that the assessment of ESS/EDS may be insufficient for predicting TC-MRBs among professional truck drivers. We assessed TC-MRBs from behavioral analysis using real collision footage, while the Bern continuous and high-resolution wake-sleep (BERN) criteria define microsleep episodes as periods of sleep with a 1–15 s duration in a driving simulation, and a combined assessment of neurophysiological, behavioral, and performance parameters^32^. These reports suggest that microsleep is behaviorally evaluable. Furthermore, many previous studies showed significant variation in the accuracy of identifying SDB using only overnight oximetry (specificity: 57.8–100.0%, sensitivity: 31.0–98.0%)^18,19^. The data indicate that screening using only overnight oximetry to identify SDB is inconsistent, whereas one study showed that the cost of home sleep testing to detect SDB was cheaper than that of PSG^33^. This finding suggests that screening for SDB using overnight pulse oximetry or home cardiorespiratory monitoring tools might minimize the cost. Therefore, a combination of methods, such as a questionnaire and overnight pulse oximetry or home cardiorespiratory monitoring tools (i.e., type-4 or type-3 PSG), as well as dashcam video footage, may be required to detect MVAs and TC-MRBs. Further study is needed to show the accuracy of the combination of methods for detecting TC-MRBs in professional drivers.

CPAP and oral appliance (OA) are widely used for the treatment of SDB in general populations. In patients with SDB, the treatment using CPAP and OA significantly reduces sleepiness, which is one of the factors predisposing to TC-MRB as mentioned above^25,26,34,35^. In agreement with the findings, CPAP treatment significantly reduces MVAs in general populations with SDB, while there has been no evidence for reduction of MVAs when using OA has treatment^36,37^. Our previous case report showed insufficient effects of treatment using OA on reducing MVAs in professional drivers, which might be due to the fact that assessment for compliance with OA treatment was subjective^38^. These findings suggest that SDB treatment using CPAP or OA with compliance assessment based on objective data would be needed to reduce TC-MRBs in the professional driver.

This study has several strengths. We reported that, in subjective statements, all the professional drivers involved in TC-MRBs fell asleep at the wheel; this was further confirmed with dashcam video footage. Since the stratification of participants into TC-MRBs groups with or without falling asleep is difficult, this dual assessment method using additional dashcam video footage provides clearer evidence regarding the occurrence of falling asleep at the wheel than subjective statements alone. An additional strength was that the drivers were employees at a large company with 400 branches across Japan. Road conditions vary according to the weather and season, especially in snowy areas; however, their effect on TC-MRBs might have been reduced in this study due to the wide distribution of drivers across the country. Nonetheless, there are limitations to consider. First, TC-MRBs were defined as involving microsleep episodes using video-based behavioral analysis, rather than diagnosing microsleep by electroencephalography. Behavioral assessment of microsleep based on dashcam video footage is possible, but its correlation with physiological assessment by electroencephalogram (EEG) recording has not been clarified. Therefore, although recording EEG while driving a truck is potentially dangerous and unrealistic, it is necessary to examine the accuracy of diagnostic methods for microsleep behaviors by simultaneously performing EEG analysis and behavioral assessment using dashcam video footage. A second limitation is that the non-TC-MRBs group included truck drivers who fell asleep at the wheel but were not involved in truck collisions. Although the drivers may have experienced near-miss cases of TC-MRBs, they could not be identified as such in the database and were potentially excluded from the non-TC-MRBs group. In addition, we could not check all video data for the non-TC-MRBs group when they were working because of the limitations in time for checking and the storage of the dashcam data. A third limitation is the possible inclusion of drivers with sleep disorders, such as narcolepsy, idiopathic hypersomnia, and insufficient sleep syndrome. The database in this study was based on regular health checks but lacked detailed sleep assessments, which could confound the study results. Fourth, we could not include sex as a variable in the logistic regression analysis since this study did not include any female drivers. Therefore, our study could not explore sex-related differences in the incidence of TC-MRBs. Finally, the results of this study would be impacted as the settings of this study utilizing a health check did not correct for the fact that professional drivers might attempt to underreport their sleepiness on questionnaires in order to maintain their driving licenses. However, since the aim of this study was to determine whether health check screening using questionnaires and overnight oximetry was useful for predicting TC-MRBs in professional drivers, we chose these methods although we recognized the limitations. Further studies are required to address these limitations and confirm our findings.

In conclusion, this study showed that the incidence of TC-MRBs was significantly associated with night-time drivers, 4% ODI, and nadir SpO_2_, but not with mean SpO_2_, ESS/EDS, and 3% ODI, all of which were verified using dashcam video footage in professional truck drivers. However, the sensitivity of overnight pulse oximetry parameters to predict TC-MRBs in a real-world application was poor. Therefore, a strategy that combines subjective and objective assessment methods, such as behavioral assessment using dashcam video footage, may be needed to achieve high accuracy in predicting TC-MRBs among professional truck drivers.

## Methods

### Subjects

The professional truck drivers included in this study belonged to a transport company with 400 branches across Japan. The inclusion criteria were (1) age ≥ 18 years and (2) employment as a truck driver at the company from April 2016 to December 2022. We excluded professional truck drivers who (1) had a health check over one year before or after the TC-MRBs; (2) had a truck collision that was not TC-MRB-related; and (3) had more than two truck collisions during the study period, as it would complicate the calculation of the period from the health check to TC-MRB due to multiple accidents. The working time zone (daytime or night-time) of the professional truck drivers remained consistent throughout the study period and they were working only at their designated working time. Daytime and night-time professional truck drivers usually worked from 8:00 AM to 7:59 PM and 8:00 PM to 7:59 AM, respectively. Professional truck drivers with severe diseases, such as heart failure, chronic obstructive pulmonary disease, or epilepsy, were excluded.

The Ethics Committee for Epidemiology of Hiroshima University approved the study protocol (approval number, #E-2814) and this study was in accordance with the tenets of the Declaration of Helsinki. Due to the retrospective nature of the study, the requirement for informed consent was waived by the Ethics Committee for Epidemiology of Hiroshima University; instead, an opt-out statement was used for consent.

### Definition of truck collisions attributed to microsleep-related behaviors

We defined TC-MRBs as collisions attributed to behavioral microsleep during their working time with the occurrence being confirmed on the dashcam video footage. In this study, the TC-MRBs group consisted of professional truck drivers who reported during interviews that their truck collisions were caused by falling asleep. After the interviews, we reviewed the 1-min dashcam video footage before the TC-MRBs to confirm that all the professional truck drivers had been involved in TC-MRBs. The dashcam video footage was recorded from the inside and outside of the truck to confirm the behavior of both the truck driver and the other vehicle’s driver. In the video footage, if the professional truck driver closed their eyes or had an absence of body movements (indicating a microsleep) for one second or more just before the collision, they were classified as being involved in a TC-MRB^23^. Using the dashcam video footage, we also assessed abnormal vehicle behavior such as inappropriate line crossing (i.e., steering of the truck over the straight or curved lines followed by recovery) that indicated the onset of a microsleep and this was regarded as having a microsleep. The control group comprised professional truck drivers who were not involved in truck collisions (non-TC-MRBs group), since this study aimed to identify solutions for reducing TC-MRBs in professional truck drivers.

### Health check

We extracted data on age, sex, body weight, body height, and blood pressure from the health check database. The BMI was calculated using the extracted data. For Asians, the World Health Organization defined a BMI ≥ 25.0 kg/m^2^ as the cutoff value for obesity^39^. We also extracted data about overnight pulse oximetry and daytime sleepiness, as scored using the ESS at the health check^40^. Regarding overnight pulse oximetry, the sleep tests were conducted at the participants’ homes using a range of oximetry devices (Pulsox-300i, Konica Minolta, Tokyo, Japan; PulSleep LS-140, Fukuda Denshi, Tokyo, Japan; PulseWatch PMP-200Gplusx, SmartWatch PMP-300EX, Philips, Best, Netherlands; ApnoMonitor7, ApnoMonitor mini, Chest Co., Tokyo, Japan; Somny, NGK Spark Plug Co., Aichi, Japan) and were manually scored at multiple facilities across Japan. The overnight pulse oximetry parameters considered in this study were the 3% ODI, 4% ODI, nadir SpO_2_, and mean SpO_2_. The 3% ODI and 4% ODI were calculated as the number of events per hour of recording time with a ≥ 3% and ≥ 4% desaturation, respectively^18^. Nadir SpO_2_ and mean SpO_2_ were defined as the lowest and mean saturation levels during the recording time, respectively. The probable severity of SDB of the professional drivers was categorized into four groups based on 3% ODI: non-SDB (< 5.0 times/h), mild SDB (5.0–14.9 times/h), moderate SDB (15.0–29.9 times/h), and severe SDB (≥ 30.0 times/h). As for subjective reports of sleepiness, the ESS was completed immediately after the sleep study to assess daytime sleepiness. The ESS consists of eight items ranging from 0 to 24 points. A higher ESS value indicates greater daytime sleepiness and the cutoff value for EDS is set at 10/11 points in our study.

### Statistical analysis

The obtained data are presented as mean ± SD, number (%), OR [95% Cl], or AUC [95% CI]. The Gaussian distribution was evaluated using the Shapiro–Wilk test, and the *t*-test or Mann–Whitney U test was used to compare continuous data. Drivers without TC-MRBs were matched with drivers with TC-MRBs using the propensity matching method (nearest neighbor method, TC-MRBs: non-TC-MRBs = 1:1, caliper: 0.20). The included variables for the matching were age and sex. For comparison, we also calculated the effect size using Cohen's d. Binary data were compared using the chi-square test. Factors associated with TC-MRBs were estimated via logistic regression analysis using a stepwise method (backward selection, removing terms with p ≥ 0.20). Variables included in the logistic regression analysis were age, BMI, working time zone, 3% ODI, 4% ODI, nadir SpO_2_, mean SpO_2_, ESS, and the presence of EDS. Regarding ODI, nadir SpO_2_, and mean SpO_2_, the OR and 95% CI of these valuables were calculated as a change of one standard deviation in the logistic regression analysis. The ROC curve with Youden’s index was used to determine the cutoff values of SDB-related parameters for TC-MRBs. We rejected the null hypothesis when the two-sided p-value was < 0.05. All statistical analyses were performed using Stata version 15.1 (Stata-Corp, TX, USA).

### Supplementary Information

Supplementary Tables.

## Data Availability

The data in this study are available from the corresponding author upon reasonable request.
