# Supplementary material for: Risk factors for collisions attributed to microsleep-related behaviors while driving in professional truck drivers
Source: Sci Rep. 2024 Mar 16;14:6378. doi: 10.1038/s41598-024-57021-1 (PMC10944504; doi:10.1038/s41598-024-57021-1)
Supplement: Supplementary file 1 — Supplementary Tables. [file 41598_2024_57021_MOESM1_ESM.docx]

**Supplementary Table 1 Detail of the number of professional truck drivers**

| **Period** | **Number** |
| --- | --- |
| April 2016-March 2017 | 14,048 |
| April 2017-March 2018 | 13,864 |
| April 2018-March 2019 | 13,861 |
| April 2019-March 2020 | 14,266 |
| April 2020-March 2021 | 14,695 |
| April 2021-March 2022 | 14,622 |
| April 2022-December 2022 | 14,613 |
| Average | 14,281 |

**Supplementary Table 2 Detail of the number of professional truck drivers with single truck collision**

| **Period** | **Number** |
| --- | --- |
| April 2016-March 2017 | 867 |
| April 2017-March 2018 | 818 |
| April 2018-March 2019 | 681 |
| April 2019-March 2020 | 602 |
| April 2020-March 2021 | 605 |
| April 2021-March 2022 | 567 |
| April 2022-December 2022 | 452 |
| Total | 4,592 |
